# Integration of new digital antenatal care tools using the WHO SMART guideline approach: Experiences from Rwanda and Zambia

**DOI:** 10.1177/20552076221076256

**Published:** 2022-02-02

**Authors:** Rosemary Muliokela, Gilbert Uwayezu, Candide Tran Ngoc, María Barreix, Tigest Tamrat, Andrew Kashoka, Caren Chizuni, Muyereka Nyirenda, Natschja Ratanaprayul, Sarai Malumo, Vincent Mutabazi, Garrett Mehl, Edith Munyana, Felix Sayinzoga, Özge Tunçalp

**Affiliations:** 1 Independent Digital Health Consultant, Lusaka, Zambia; 2 Thousand Hill Solutions, Kigali, Rwanda; 3 World Health Organization, Rwanda Country Office, Kigali, Rwanda; 4 UNDP/UNFPA/UNICEF/WHO/World Bank Special Programme of Research, Development and Research Training in Human Reproduction (HRP), Department of Sexual and Reproductive Health and Research, 3489 World Health Organization, Geneva, Switzerland; 5 Ministry of Community Development and Social Services—Information, Communication, Technologies (ICT), Zambia, Lusaka, Zambia; 6 108232 Ministry of Health Zambia, Maternal Health Unit, Lusaka, Zambia; 7 World Health Organization, Zambia Country Office, Lusaka, Zambia; 8 3489 World Health Organization, Department of Digital Health and Innovations, Geneva, Switzerland; 9 108022 Ministry of Health Rwanda, Kigali, Rwanda; 10 108022 Ministry of Health Rwanda, Maternal, Child, and Community Health, Kigali, Rwanda

**Keywords:** Digital health, guidelines, antenatal care, country adaptation, quality of care

## Abstract

**Objectives:**

Digital tools for decision-support and health records can address the protracted process of guideline adoption at local levels and accelerate countries’ implementation of new health policies and programmes. World Health Organization (WHO) launched the SMART Guidelines approach to support the uptake of clinical, public health, and data recommendations within digital systems. SMART guidelines are a package of tools that include Digital Adaptation Kits (DAKs), which distill WHO guidelines into a format that facilitates translation into digital systems. SMART Guidelines also include reference software applications known as digital modules.

**Methods:**

This paper details the structured process to inform the adaptation of the WHO antenatal care (ANC) digital module to align with country-specific ANC packages for Zambia and Rwanda using the DAK. Digital landscape assessments were conducted to determine potential integrations between the ANC digital module and existing systems. A multi-stakeholder team consisting of Ministry of Health technical officers representing maternal health, HIV, digital health, and monitoring and evaluation at district and national levels was assembled to review existing guidelines to adapt the DAK.

**Results:**

The landscape analysis resulted in considerations for integrating the ANC module into the broader digital ecosystems of both countries. Adaptations to the DAK included adding national services not reflected in the generic DAK and modification of decision support logic and indicators. Over 80% of the generic DAK content was consistent with processes for both countries. The adapted DAK will inform the customization of country-specific ANC digital modules.

**Conclusion:**

Both countries found that coordination between maternal and digital health leads was critical to ensuring requirements were accurately reflected within the ANC digital module. Additionally, DAKs provided a structured process for gathering requirements, reviewing and addressing gaps within existing systems, and aligning clinical content.

## Introduction

**Figure 1.**
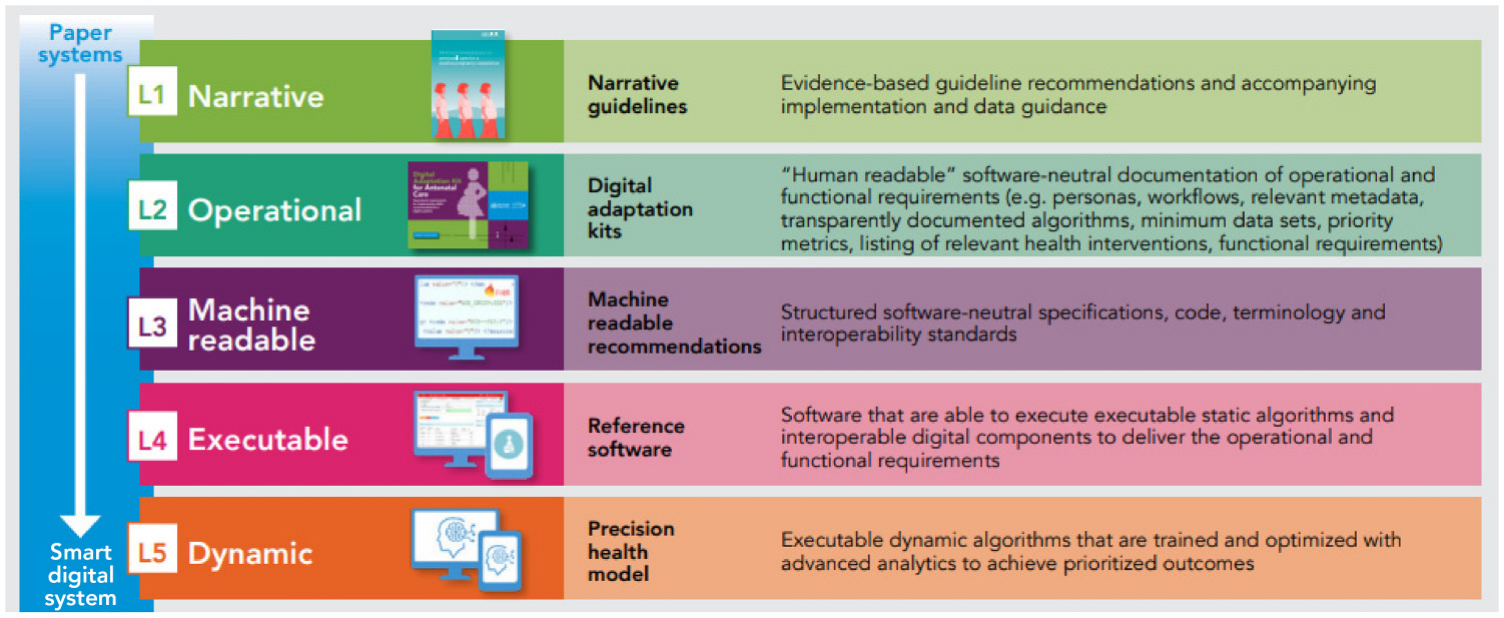
Overview of the SMART guidelines components, in which digital adaptation kits guided the country customization of the reference software.^2,4^

As countries embark on digitalizing the health sector to achieve universal health coverage,^1^ the World Health Organization (WHO) launched the SMART—standards-based, machine-readable, adaptive, requirements-based, and testable—guidelines approach to optimize the adoption of WHO guidelines through digital systems. SMART guidelines are a package of tools that include Digital Adaptation Kits (DAKs), which detail the generic health workflows, core data elements, and decision logic derived from WHO guidelines as a starting point for incorporating into digital systems.^2,3^ Additionally, SMART Guidelines include reference software modules derived from WHO guidelines (see Figure 1).^2^

In parallel, since 2018, the Ministries of Health (MOH) of Zambia and Rwanda, with support from WHO, initiated the adaptation of the 2016 recommendations on antenatal care (ANC) for a positive pregnancy experience, to provide pregnant women with respectful, individualized, person-centred care at every contact.^5^ This process led to the updating of national ANC guidelines and the design of an implementation research study to strengthen the quality of an integrated country-specific ANC service package (including malaria, HIV, TB). The study aims to test innovative ANC service delivery approaches and mechanisms and has two phases: formative and demonstration. The formative phase seeks to adapt the WHO digital ANC module, a person-centric digital health record and decision support tool for healthcare providers,^6^ to the country-specific ANC service delivery package. The adapted Zambia and Rwanda ANC digital modules will be user-tested by healthcare workers for usability and improvements, prior to evaluating the country-specific ANC package and the tailored digital ANC module in selected districts.

Rwanda and Zambia both have well-established digital health ecosystems, with increasing demand and penetration of digital health services over the past decade. This has also resulted in fragmented digital health landscapes, characterized by numerous pilot projects, resulting in silos with significant barriers to accessing and sharing data for decision and policymaking. Additionally, inadequate standards and legislation for digital health have hindered the collection, processing, and sharing of health data and limited continuity of care. In view of these challenges, both countries have developed digital health strategies to improve service delivery and patient experience.^7,8^

In preparation for the formative phase, both countries conducted landscape analyses to customize the generic ANC digital module through a review of the ANC DAK. The ANC DAK details the underlying health and data content of the ANC module and provides the metadata and decision-support logic that can be applied across a variety of digital systems.^4^ The objective of this article is to document the customization requirements for the generic WHO digital ANC module to the Rwandan and Zambian contexts through the use of the DAK and provide considerations for integration between existing systems and the digital module. Under the SMART guideline approach by WHO, the use of the DAKs to guide country customization represents a unique and systematic approach to ensure that countries’ digital systems are verified and aligned to national service delivery packages and WHO's evidence-based recommendations. Furthermore, this paper identifies lessons for future adoption of WHO SMART Guidelines at the country level.

## Methods

The landscape assessment was conducted with the support of consultants in both Rwanda (GU) and Zambia (RM). This process included a desk review of reference documents, landscape analysis of digital systems, and validation by respective MOH staff. The consultants also held a series of consultations and interviews with key stakeholders in the digital health ecosystem. This work also entailed convening critical government stakeholders involved in digital health policy and implementation to map the roles of different actors. Additionally, the country teams comprised of the MOH, WHO country offices and consultants, explored various digital health strategic priorities and status of implementations to identify synergies and alignment with the WHO digital ANC module.

The digital health landscape assessments were fundamental to determining how the country-adapted versions of the ANC digital module would be integrated ‘to support a cohesive approach to implementation, in which different digital interventions can operate together, rather than as duplicative and isolated implementations.’^9^ In both countries, it was critical to identify and map the specific areas where the module could add value and ascertain the scale of deployment to complement existing digital health systems.^10,11^ These included SmartCare, the national electronic medical record (EMR) for Zambia, which contains a maternal and child health (MCH) module, and the Rwanda EMR (OpenMRS), RapidPro, and national health management information system (DHIS2).^10,11^ The analysis further highlighted the potential integration points and considerations for the adaptation for both country contexts.

The limited availability of documentation to describe the architecture and interoperability framework of existing digital systems proved to be a challenge. However, this was resolved through the MOH digital health team engaging partners and software developers who had supported the development of those systems. Further, not all systems on maternal health were adequately documented with data dictionaries and business rules. However, through stakeholder engagements with appropriate program and digital health leads, missing information was gathered and used to inform the assessment.

## Results

In both Zambia and Rwanda, the landscape analysis detailed the digital transformation process, digital health strategic priorities, and existing digital implementations related to ANC. Outcomes from the landscape assessment were grouped according to the WHO classifications of digital health interventions.^12^

Considerations and recommendations for digital integration were guided by existing digital health governance frameworks in both countries. In Zambia, a MOH committee reviews every new digital tool entering the ecosystem to ensure adherence to national standards, including interoperability and data privacy standards. Private-sector technology partners are also critical to this process as they are key stakeholders for implementation to ensure the process of software development and deployment adheres to national digital governance standards. The following are key recommendations and considerations for the adaptation of the WHO digital ANC module and integrating it into the digital health ecosystem of both countries:

- Data infrastructure requirements of the module would need to be developed using existing guidelines and policies on data access, hosting (e.g. local, cloud), data interoperability and interactions with the national data warehouse. This would require a mapping exercise of systems against business requirements and reporting needs.
- Integrations of the ANC module will need to occur via Application Programming Interface, a software intermediary to interact with systems such as SmartCare, DHIS2 and RapidPro that are already capturing and storing ANC data.^10,11^
- IT infrastructure and skills gap assessment should be conducted prior to system deployment to ensure optimal usage of the tool.
- Other considerations for integration included: the development of an implementation plan with continuous stakeholder engagement to build ownership of the process, as well as the selection and identification of a local technical partner to adapt and develop the software to the local context.

### Identifying customization requirements for ANC module by adapting the DAK

The country customization needs of the WHO digital ANC module was guided through review of the ANC DAK. Both countries setup DAK adaptation teams, which comprised of stakeholders within the digital health ecosystem, including information and communications technology (ICT), MCH, reproductive health, and monitoring & evaluation units in the MOH, and other development partners. A series of stakeholder meetings were held to align requirements with DAK components, including gaining consensus on how to integrate the module with existing ANC tools and systems, such as HMIS, SmartCare, Rwandan EMR, and RapidPro.^10,11^ The meetings also provided an overview of existing systems, identified gaps in line with updated country ANC guidelines, and mapped requirements for improvements and potential integrations of the digital module. Decisions on modifications and inclusions into the adapted module were reached through consensus by relevant stakeholders based on national protocols and guidelines. Further analysis of these decisions was based on the adapted ANC package; the MOH validated and approved the final consensus and decisions.

Each component of the generic DAK was reviewed in virtual and in-person stakeholder meetings, with both maternal health and ICT teams to verify and align to the local context. Over 80% of the content from the generic ANC DAK was adopted as it was consistent with the country processes for both countries. However, country teams also added, removed, and modified elements from the DAK in accordance with national requirements and service delivery packages. For example, the Zambia team expanded on the data fields and decision-support logic for the prevention of mother-to-child transmission, which were derived from the Zambia Consolidated Guidelines for Treatment and Prevention of HIV.^13^ For Rwanda, the decision support logic expanded on the set of danger signs and modified immunization schedules; the country team also included additional ANC quality indicators and data elements for context-specific services, such as mosquito bed net distributions. Across both sites, registration-related data fields were modified to account for national identification numbers, location hierarchies, and related administrative requirements.

Other DAK components, such as the user scenarios and personas, required interviews and observations at health facilities. Data for these were collected at health facilities selected as implementation research sites. During each stakeholder meeting, a consensus on requirements and content was reached. The consensus was informed by relevant literature and practice in the local context. Additionally, functional requirements were enhanced through user feedback. For example, the inclusion of referral facilities’ phone numbers and ability to make calls within the system were added to the functional requirements in order to strengthen the coordination of ANC referrals and ensure pregnant women’s timely access to services. Some non-functional requirements included ensuring user-friendliness of the system to accommodate health workers with low digital literacy skills. Further, as the study will be conducted across multiple facilities at the primary healthcare level, the system should allow for data exchange and efficient synchronization across multiple facilities.

## Conclusion

Identifying the customization requirements of the ANC digital module through the DAK provided an opportunity to strengthen the content within the country-customized module, as well as support other digital systems to align with the current WHO clinical recommendations and digital health standards. The adapted DAKs have since been transferred to local technology teams who will execute the required customizations of the generic WHO ANC module to country contexts. This example represents the first effort for the DAK and ANC module to be used together to inform national development processes of digital tools. The experience from Rwanda and Zambia will be instrumental for other countries looking to utilize the DAK approach to digitize health services. Furthermore, the process of integrating different digital systems varies across countries and depends on the national standards and policies for interoperability and the maturity of the digital health ecosystem. The implementation experiences from the digital integration of the ANC digital module with national EMR systems will be documented in the forthcoming implementation research phase and will contribute to learnings for other countries’ initiatives conducting similar processes of integrating different digital systems. Although we use ANC as an example to illustrate the adaptation of the digital module using the DAK, this process of customizing SMART guideline components will be relevant for other health domain areas to ensure integrated health system strengthening.

Overall, this process demonstrated that strong leadership, governance, and coordination are key elements for the design of digital health implementations in any setting. It is critical to align any new digital tool with existing digital governance systems to ensure local ownership. Collaboration and constant engagement from the project outset between maternal program and ICT leads was very critical to coordinate requirements for the adaptation of the generic WHO ANC digital module in both countries. As the SMART guidelines, including the DAKs, are a new approach, it will be important to capacitate Ministries of Health to ensure ownership of the adaptation processes from the outset.

## Supplemental Material

sj-docx-1-dhj-10.1177_20552076221076256 - Supplemental material for Integration of new digital antenatal care tools using the WHO SMART guideline approach: Experiences from Rwanda and Zambia Click here for additional data file. Supplemental material, sj-docx-1-dhj-10.1177_20552076221076256 for Integration of new digital antenatal care tools using the WHO SMART guideline approach: Experiences from Rwanda and Zambia by Rosemary Muliokela, Gilbert Uwayezu, Candide Tran Ngoc, María Barreix, Tigest Tamrat, Andrew Kashoka, Caren Chizuni, Muyereka Nyirenda, Natschja Ratanaprayul, Sarai Malumo, Vincent Mutabazi, Garrett Mehl, Edith Munyana, Felix Sayinzoga and Özge Tunçalp in Digital Health
