# Supplementary material for: Integration of new digital antenatal care tools using the WHO SMART guideline approach: Experiences from Rwanda and Zambia
Source: Digit Health. 2022 Feb 2;8:20552076221076256. doi: 10.1177/20552076221076256 (PMC8814973; doi:10.1177/20552076221076256)
Supplement: sj-docx-1-dhj-10.1177_20552076221076256 - Supplemental material for Integration of new digital antenatal care tools using the WHO SMART guideline approach: Experiences from Rwanda and Zambia [file sj-docx-1-dhj-10.1177_20552076221076256.docx]

**Title**: Integration of new digital antenatal care tools using the WHO SMART guideline approach: Experiences from Rwanda and Zambia

**Short title:** Digital antenatal care tools using SMART Guidelines

**Supplement 1. More information on WHO ANC Module and Digital Adaptation Kit**

This supplement provides screenshots of the WHO ANC Module to illustrate the key components of this digital tool. The Digital Adaptation Kit details the data and decision-support content underlying the digital module to facilitate country adaptation of the generic version of the ANC module.

**Figure 1. Screenshots of the WHO ANC module**


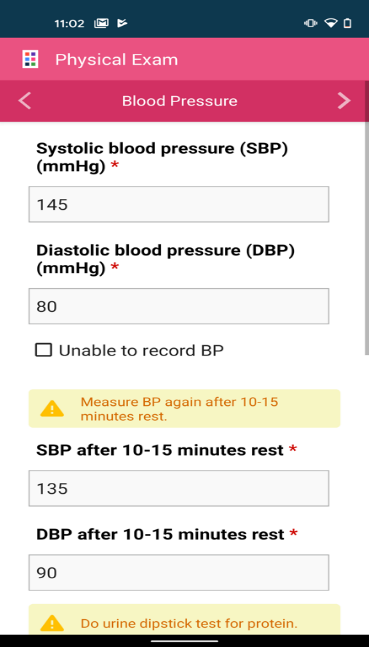

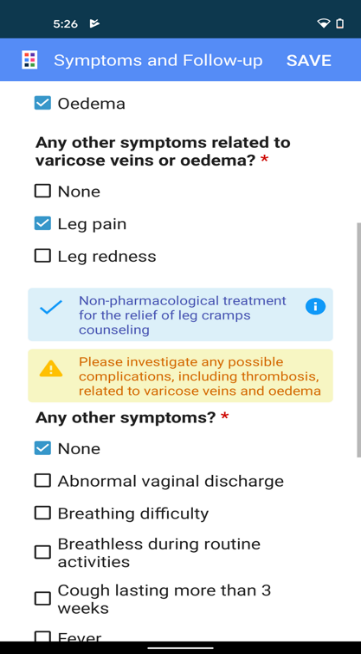

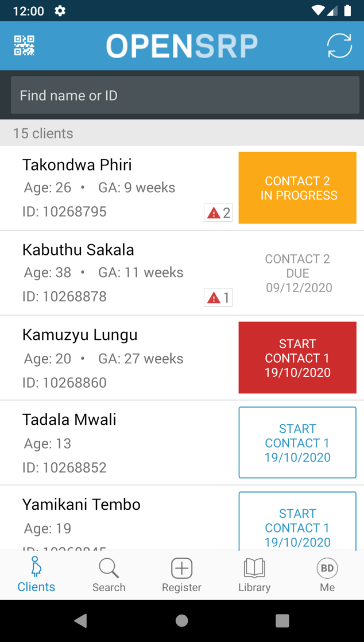

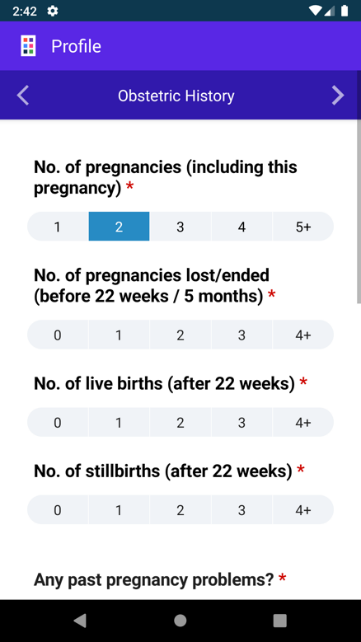

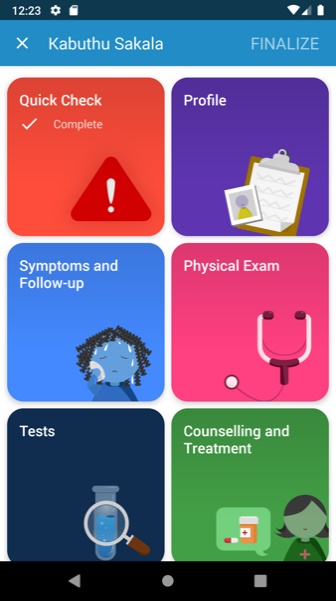

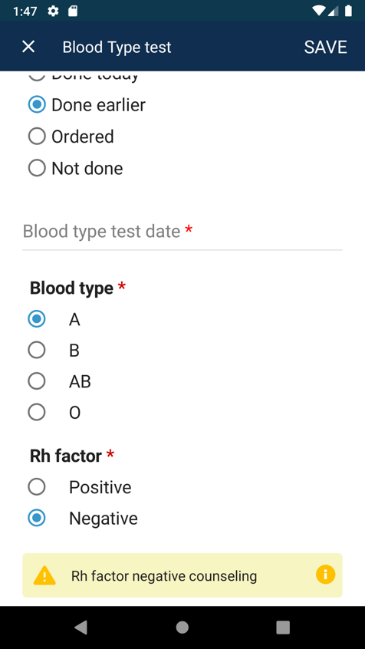

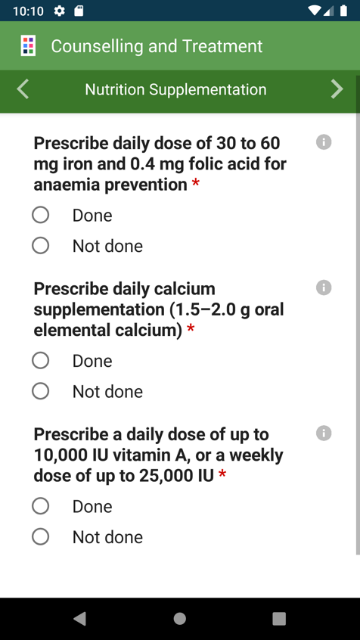

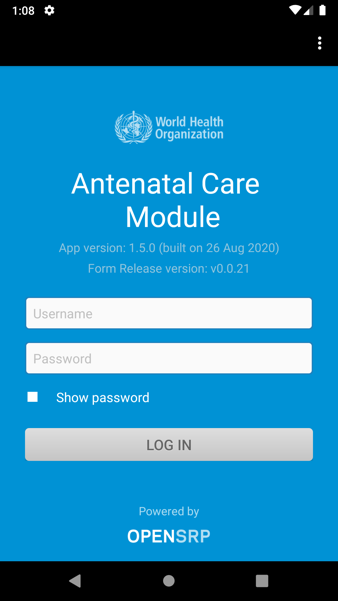


**Figure 2. Data dictionary component of the DAK corresponding to the content in ANC digital module**


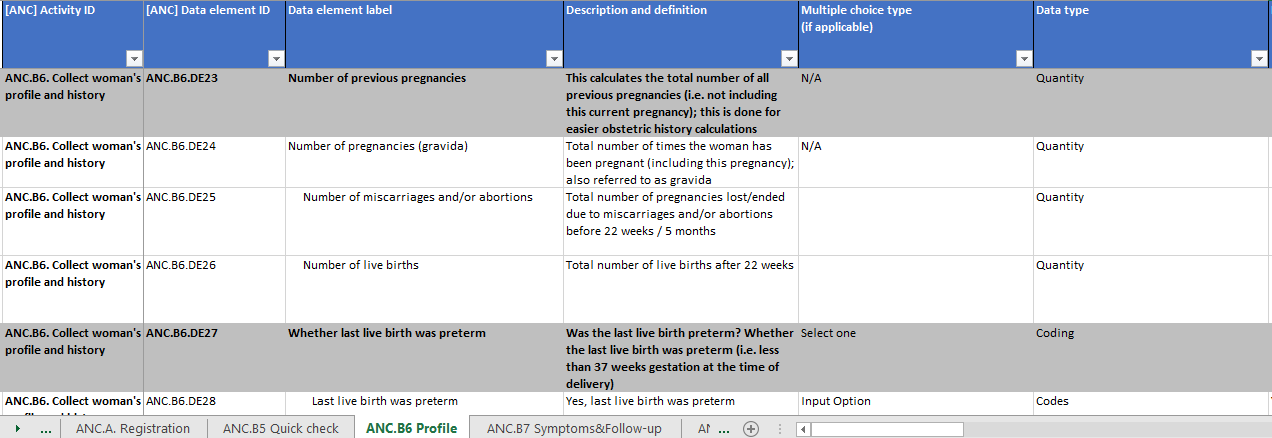


**Figure 3. Decision-support logic component of the DAK embedded within the ANC digital module
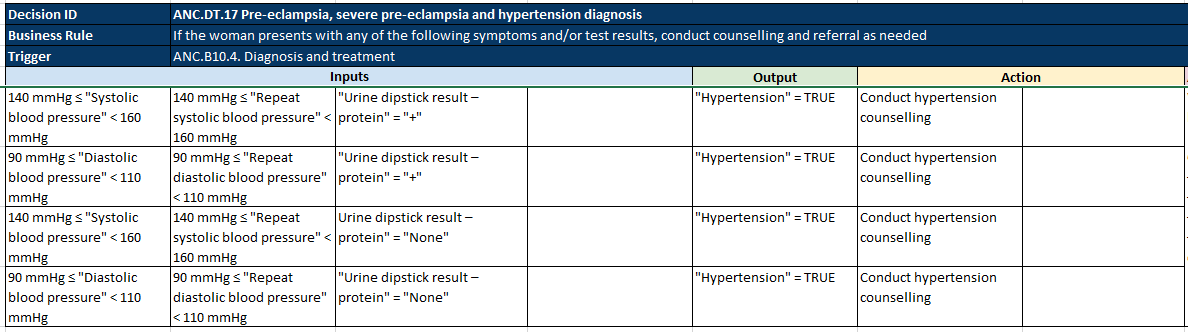
**
